# The impact of low-energy phonon lifetimes on the magnetic relaxation in a dysprosocenium single-molecule magnet†

**DOI:** 10.1039/d4cc03768e

**Published:** 2024-10-23

**Authors:** Rizwan Nabi, Benjamin E. Atkinson, Jakob K. Staab, Jonathan M. Skelton, Nicholas F. Chilton

**Affiliations:** a Department of Chemistry, The University of Manchester Manchester M13 9PL UK jonathan.skelton@manchester.ac.uk; b Research School of Chemistry, The Australian National University Canberra ACT 2601 Australia nicholas.chilton@anu.edu.au

## Abstract

Developing molecular spin technologies requires microscopic knowledge of their spin-dynamics. Calculation of phonon modes, phonon scattering and spin–phonon coupling for a dysprosocenium single-molecule magnet (SMM) give simulations of spin-dynamics that agree with experiment. They show that low-energy phonon scattering is a significant contribution to the high-performance of dysprosocenium SMMs.

## Abstract 2

Calculation of phonons, phonon scattering, spin–phonon coupling and magnetic relaxation rates for a dysprosocenium single-molecule magnet show that strong scattering of low-energy modes is a significant contribution to their high-performance.

## Graphical abstract

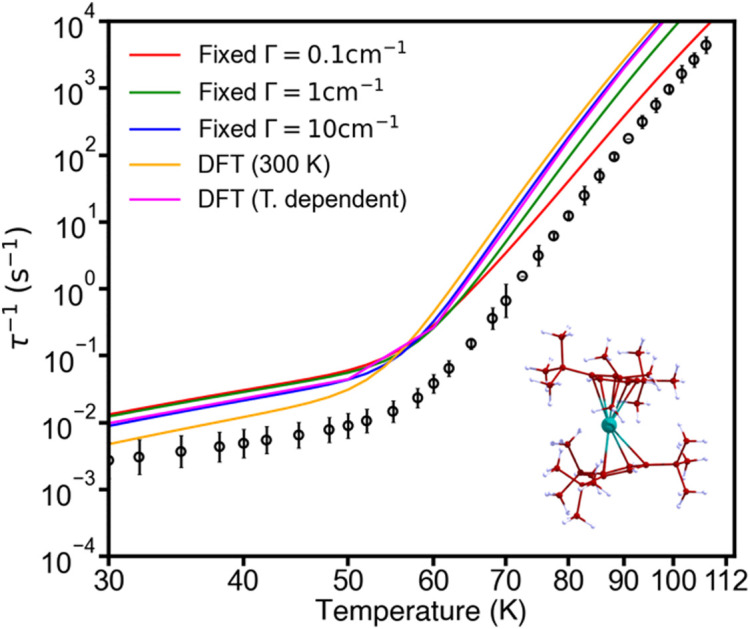

Molecular spins are a powerful platform for exploring the limits to which we can build the smallest possible arbitrarily-configurable magnetic materials, with applications to high-density data storage and quantum computing.^1,2^ A fundamental challenge in the area of SMMs is to raise the maximum temperature at which they exhibit magnetic memory. Currently, the highest temperature at which magnetic hysteresis has been observed is 80 K, in two Dy(iii)-based SMMs, *viz.* [(Cp^iPr5^)Dy(Cp*)][B(C_6_F_5_)_4_] and [(Cp^iPr5^)DyI_3_Dy(Cp^iPr5^)], with fully substituted cyclopentadienyl ligands (Cp^iPr5^ = C_5_(CH(CH_3_)_2_)_5_, Cp* = C_5_(CH_3_)_5_).^3,4^ In both systems, the pseudo-linear disposition of the cyclopentadienyl anions creates large axial magnetic anisotropy, and [(Cp^iPr5^)DyI_3_Dy(Cp^iPr5^)] also features strong exchange coupling arising from a 5d half σ-bond between the two Dy(iii) ions, which is thought to be the origin of its ultrahard magnetic coercivity. Since the magnetic relaxation (*i.e.* loss of magnetic memory) is driven by spin–phonon coupling through two-phonon Raman scattering and single-phonon Orbach mechanisms, a crucial component of improving performance is understanding the spin–phonon coupling in SMMs.^5–12^ The Orbach mechanism is a single-phonon process whose characteristic time has an exponential temperature dependence 
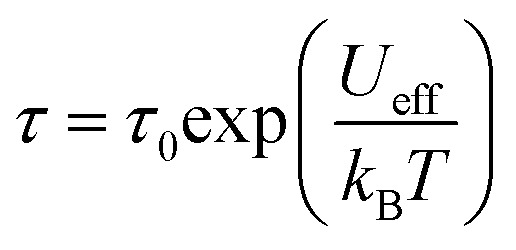
, where *τ*_0_ is the characteristic time scale of the phonon bath and *U*_eff_ is the energy barrier.^13^ At high temperature, SMMs reverse their magnetisation over the *U*_eff_ barrier *via* scattering with high-energy optical phonons, but this mechanism is suppressed at low temperatures when the population of these phonons is limited. At lower temperatures, magnetic relaxation is instead dominated by the two-phonon Raman-I process, driven by scattering with low-energy (pseudo-)acoustic phonons.^5,6,9,10^ Understanding the nature of the phonons in molecular crystals and the interplay of these mechanisms is thus crucial to controlling magnetic relaxation in SMMs.

To this end, we have developed open source software packages to perform *ab initio* calculations of spin–phonon magnetic relaxation dynamics in the solid state,^10,11,14^ and have demonstrated excellent agreement with experiments for Dy(iii) SMMs in the crystalline^11^ and amorphous solid phases.^15^ Here we deploy our methods (see SI for details) to study the magnetic relaxation of the first dysprosocenium SMM [DyCp^ttt^_2_][B(C_6_F_5_)_4_] (1; Cp^ttt^ = 1,2,4-(C(CH_3_)_3_)–C_5_),^16^ including *ab initio* calculation of the phonon linewidths. We find that strong phonon scattering at low temperature leads to shorter than expected phonon lifetimes, and hence larger phonon linewidths, which partially contributes to the suppression of Raman relaxation at low temperature. This suggests a possible route to slowing two-phonon Raman spin-dynamics in molecular materials by enhancing the low-energy phonon scattering.

The crystal structure of 1 has the *P*1̄ space group and contains a monometallic dysprosocenium cation [Dy(Cp^ttt^)_2_]^+^ and a charge-balancing perfluorinated [B(C_6_F_5_)_4_]^−^. The structure used here differs from the original in that it does not contain any solvent of crystallisation,^17^ but we note that the experimental magnetic relaxation rates for the pure crystalline material are practically identical for the two forms, showing single-phonon Orbach relaxation above 60 K, two-phonon Raman-I relaxation between 50 and 30 K, and quantum tunnelling of the magnetisation (QTM) below 20 K.^17^

The asymmetric unit contains one formula unit (Fig. 1a), and thus there are two formula units in the primitive unit cell due to the inversion symmetry of the *P*1̄ space group (Fig. 1b), with a total of 276 atoms. Optimisation of the crystal structure of 1 using density-functional theory (DFT; see ESI† for details) gives unit cell parameters are very similar to the experimental ones (Table S1, ESI†). Calculation of the phonon spectrum of 1 with phonopy^18^ contains one small-magnitude imaginary mode at off-Γ *q*-points (Fig. 2a). This is likely an artefact of the Fourier interpolation used to obtain frequencies at *q*-points not commensurate with the 2 × 2 × 1 supercell expansion (1104 atoms), but larger expansions were not computationally viable. The low-energy phonon dispersion and phonon density of states (pDOS) shows a dense band of dispersive modes up to *ca.* 100 cm^−1^ arising from the pseudo-acoustic modes (external molecular modes, Fig. 2). We previously found that a 5 × 5 × 5 *q*-point mesh in reciprocal space was required to converge the integral over the first Brillouin zone when modelling phonon-driven magnetic relaxation.^11^ In this case, our 2 × 2 × 2, 3 × 3 × 3, 4 × 4 × 4 and 5 × 5 × 5 *q*-point meshes include 4, 14, 30 and 48 imaginary modes, respectively, with frequencies of at most 10.4*i* cm^−1^.

**Fig. 1:**
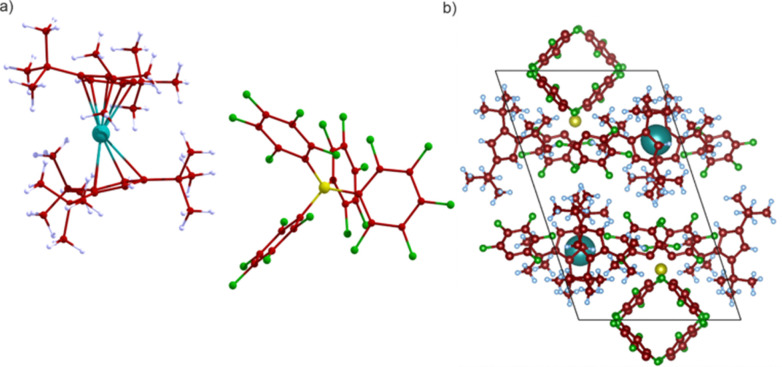
(a) Molecular structure of 1. (b) Optimised geometry of the primitive cell containing two molecules. Dy = teal, B = yellow, F = green, C = brown, H = light blue.

**Fig. 2:**
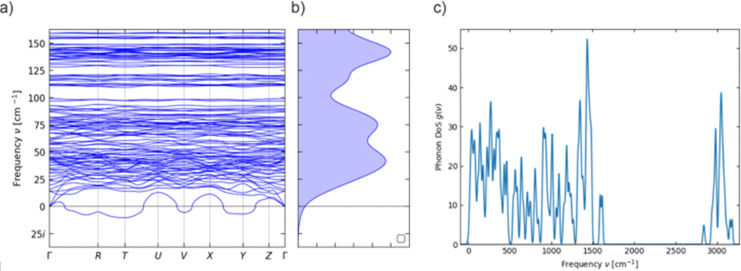
Calculated (a) low-energy phonon dispersion and (b) density of states (pDOS), and (c) full pDOS, of 1.

To test whether the latter are sufficiently converged, we calculated the spin–phonon coupling and subsequently magnetic relaxation rates of 1 using our established protocols^10,11,19^ based on state-average complete active space self-consistent field spin–orbit (SA-CASSCF-SO) calculations (see ESI† for details; note that our calculations exclude temperatures <30 K, as at lower temperatures QTM dominates). For *q*-point meshes from 1 × 1 × 1 to 5 × 5 × 5 with a fixed full-width-at-half-maximum (FWHM) phonon linewidth of 10 cm^−1^, the rates are identical (Fig. S3, ESI†). For the 4 × 4 × 4 *q*-point mesh with 30 imaginary frequencies, we tested both neglecting the imaginary modes and setting the frequencies to the absolute values, finding no differences (Fig. S4, ESI†). As such, we can be confident that calculations on 2 × 2 × 2 and 3 × 3 × 3 meshes are converged with respect to Brillouin zone integration in this case.

Compared to the experimental relaxation rates, the rates calculated using the fixed linewidth of *Γ* = 10 cm^−1^ are overestimated across the entire temperature range, but by less than an order of magnitude. Variation of the linewidth from *Γ* = 0.1 to 10 cm^−1^ shows a positive correlation with relaxation rate in the Orbach regime and a negative correlation in the Raman-I regime for all of the *q*-point meshes tested (Fig. S5–S11, ESI†). We observed and briefly discussed the latter effect previously,^11^ but give a detailed explanation of this effect in the ESI† (Fig. S12 and S13, ESI†). Comparison to the experimental data may suggest that the higher-energy optical phonons that drive Orbach relaxation should have a narrower linewidth (*Γ* ~ 0.01 cm^−1^), and the lower-energy (pseudo)acoustic phonons that drive Raman-I relaxation should have a broader linewidth (*Γ* ~ 100 cm^−1^). While this trend in agreement with DFT calculations of the phonon linewidths (see below), we note that this could also arise from an underestimation of the phonon frequencies.^20^ We also considered the thermodynamic NVT approximation for the linewidths introduced by Lunghi *et al.*:^5^ in both the Orbach and Raman-I regimes this expression gives rates very similar to those obtained using a fixed *Γ* = 10 cm^−1^ (Fig. S5–S11, ESI†), however at higher temperatures the linewidths for the very low energy phonons, which are crucial in the Raman-I regime, become so large that they cause numerical instabilities in the integrals. We take the opportunity to compare the present results to the Raman-I calculations for this compound in the original report,^16^ and those more recently performed by Lunghi.^9^ The first calculations have a vastly different temperature-dependence to the ones reported here primarily because those calculations employed only gas-phase vibrational modes and included none of the low-energy acoustic and pseudo-acoustic modes required to accurately model the Raman-I mechanism, amongst other minor methodological differences. The more recent results by Lunghi (which are closer to experiment in the Orbach region than our present results) differ in that we: employ a more realistic anti-Lorentzian lineshape (*cf.* Gaussian); include an electrostatic model of the crystalline environment (*cf.* gas-phase); account for phonon dispersion by integration the first Brillouin zone (*cf.* gamma-point only); and employ analytical derivatives of the total *ab initio* Hamiltonian to obtain the spin–phonon coupling (*cf.* numerical derivatives of crystal field parameters).

To calculate the phonon lifetimes and linewidths from first principles requires the three-phonon scattering rates to be determined using the third-order (anharmonic) force constants.^21^ Unlike our previous work on the Dy(iii) SMM [Dy(bbpen)Br],^11^ compound 1 is much lower symmetry and has more atoms in the unit cell, which makes the calculations far more demanding. We therefore calculated the third-order force constants using phono3py^21^ at different interatomic cutoff distances (3 < *r*_cut_ < 7 Å, see ESI† for details), and determined the phonon lifetimes and linewidths using 2 × 2 × 2 and 3 × 3 × 3 *q*-point meshes (note that calculated linewidths are not meaningful for imaginary modes, so these modes are excluded from rate calculations). The phonon lifetimes at 300 K range from 0.01 to 100 ps, which correspond to linewidths of ~500 to 0.05 cm^−1^ (Fig. 3 and Fig. S14, ESI†). As the cutoff is reduced from the largest *r*_cut_ = 7 Å for the smaller 2 × 2 × 2 *q*-mesh, the average difference in the calculated lifetimes at 300 K increases from 0.07 ps (4%) with *r*_cut_ = 6.5 Å to 0.65 ps (58%) with *r*_cut_ = 3 Å (Table S3, ESI†). However, given the orders of magnitude variation in the phonon lifetimes, these averages are not a meaningful metric, and a more useful test is the calculated magnetic relaxation rates.

**Fig. 3:**
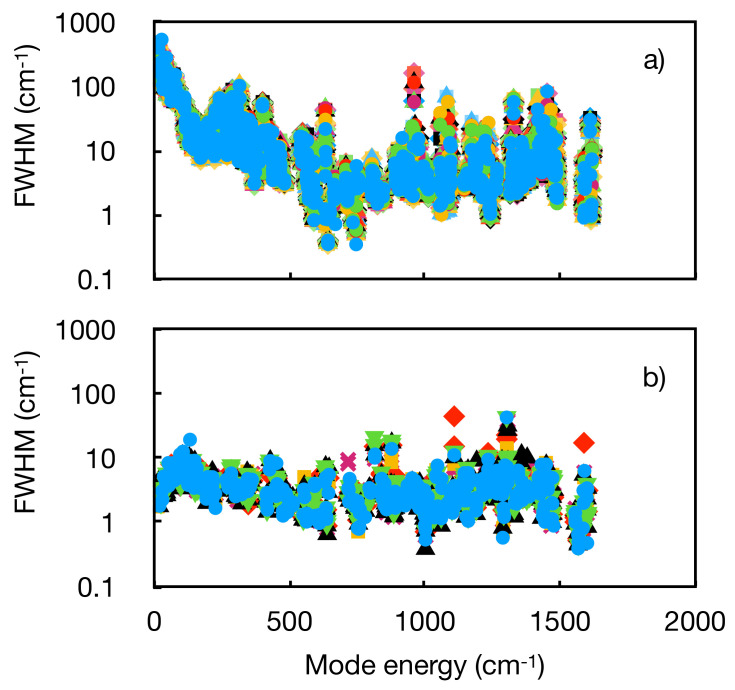
Phonon linewidths as a function of mode energy (<2000 cm^−1^ only) at unique *q*-points for (a) compound 1 with a 3 × 3 × 3 sampling mesh at 300 K (*r*_cut_ = 7 Å), and (b) [Dy(bbpen)Br] with a 2 × 2 × 2 sampling mesh at 300 K.^11^ Values for different unique *q*-points are shown by different markers and colours.

Calculating the rates using the DFT-calculated phonon linewidths at 300 K as a function of *r*_cut_, for the 2 × 2 × 2 *q*-point mesh, shows that the single-phonon Orbach rates are not greatly influenced by *r*_cut_ and are similar to the fixed *Γ* = 10 cm^−1^ calculation (Fig. S15b, ESI†). On the other hand, the two-phonon Raman-I rates show a decrease with increasing *r*_cut_, approaching the experimental rates at *r*_cut_ = 7 Å (Fig. S15a, ESI†). However, if we instead use the calculated phonon linewidths at each temperature for which we calculate magnetic relaxation rates, *i.e.* rather than using the 300 K linewidths at all temperatures, we find that neither the Orbach nor Raman-I rates show a significant dependence on the *r*_cut_ (Fig. S16, ESI†). Because there is a small dependence of the Raman rates on *r*_cut_ when phonon lifetimes are evaluated at 300 K, but no such dependence on *r*_cut_ when they are evaluated <60 K, we suggest this implies that the phonon interactions among the low-energy modes are long-ranged at high temperature but become more localised at low temperature. This is also observed as an increasing divergence in the calculated linewidths as a function of temperature for different *r*_cut_ for a low-energy mode (Fig. S18a, ESI†). This is likely a consequence of the amplitude (mean-square displacement) of the low-energy modes being strongly dependent on temperature. The same conclusions hold for the rates calculated with *r*_cut_ = 7 Å and the larger 3 × 3 × 3 *q*-point mesh (Fig. 4 and Fig. S17, ESI†), indicating our calculations are converged with respect to *q*-point mesh and *r*_cut_.

**Fig. 4:**
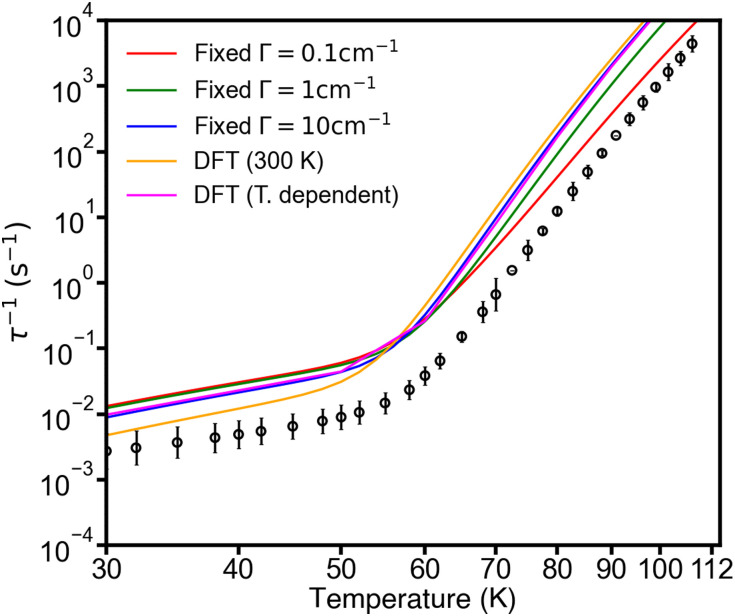
Experimental (black circles) and calculated (solid lines) magnetic relaxation rates of 1. The calculations are performed on the 3 × 3 × 3 *q*-point mesh with different approximations for the linewidth *Γ*, *viz.* fixed *Γ* = 0.1, 1 and 10 cm^−1^ (red, green and blue respectively), and DFT-calculated linewidths at 300 K (orange) and temperature dependent DFT linewidths (pink). DFT-calculated linewidths obtained with *r*_cut_ = 7 Å.

While there are significant temperature dependencies for the calculated phonon linewidths, especially at low energies (Fig. S18, ESI†), we find that when accounting for this temperature dependence the rates are very similar to those obtained at a fixed *Γ* = 10 cm^−1^, and are overestimated compared to experiment (Fig. S16 and S17, ESI†). This could suggest that the effective linewidths (lifetimes) are underestimated (overestimated) at low temperature, a suggestion we previously posed for [Dy(bbpen)Br],^11^ which may be due to calculations for an infinite perfect crystal compared to a real finite crystal with defects. However, there could be other errors in our method such as inaccurate phonon energies from DFT.^20^ In the present case for 1, we observe that the (pseudo-)acoustic modes show a trend of increasing linewidth with decreasing energy, which is the opposite to what was observed for [Dy(bbpen)Br], which showed decreasing linewidths with decreasing energy (Fig. 3).^11^ Indeed, the phonon linewidths for the lowest-energy modes in 1 approach 600 cm^−1^ at 300 K, which are an order of magnitude larger than the linewidths of *ca.* 2 cm^−1^ found for [Dy(bbpen)Br].^11^ While these behaviours are quite distinct from each other, they are also quite different to the experimentally-determined temperature dependence of phonon linewidths for [VO(acac)_2_], which range from a few cm^−1^ to 10–20 cm^−1^ at 300 K.^22^ This suggests that chemistry can play important roles in phonon dynamics beyond manipulation of phonon energies alone. Remarkably, the extreme difference in rates at 30 K (factor of 10^4^) and ∼*T*^2^*vs.* ∼*T*^4^ temperature dependence of the Raman-I relaxation in 1 and [Dy(bbpen)Br] are accurately captured by our calculations. The size of the Raman exponent has been shown to be related to the shape of the low-energy phonon DoS,^23^ and thus stronger low-energy phonon scattering giving broader phonon linewidths in 1*vs*. [Dy(bbpen)Br] must be partially responsible for the suppressed Raman-I relaxation in 1. This in turn suggests that enhancing phonon scattering may provide a route to extrinsic enhancement of the spin lifetimes at low temperature. However, the route to achieve this is unclear: measurements for **1** in an amorphous frozen solvent matrix seem to have no impact on spin lifetimes.^15,16^ We hope that the present results motivate more experimental measurements of phonon lifetimes in molecular crystals.

We thank the ERC (STG-851504), The Royal Society (URF191320) and UKRI (MR/T043121/1), and the Computational Shared Facility at The University of Manchester. This research was undertaken with the assistance of resources and services from the National Computational Infrastructure (NCI), which is supported by the Australian Government. We thank the HEC Materials Chemistry Consortium (EPSRC EP/R029431, EP/X035859) for access to the ARCHER2 UK National Supercomputing Service. NFC and JMS conceived the project. RN and BEA performed calculations. JKS and NFC developed code.

## Data availability

Research data for this publication is available *via* FigShare at: https://doi.org/10.48420/26378074.

## Conflicts of interest

There are no conflicts to declare.

## Supplementary Material

CC-060-D4CC03768E-s001

## References

[cit1] Wasielewski M. R., Forbes M. D. E., Frank N. L., Kowalski K., Scholes G. D., Yuen-Zhou J., Baldo M. A., Freedman D. E., Goldsmith R. H., Goodson T., Kirk M. L., McCusker J. K., Ogilvie J. P., Shultz D. A., Stoll S., Whaley K. B. (2020). Nat. Rev. Chem..

[cit2] Chilton N. F. (2022). Annu. Rev. Mater. Res..

[cit3] Guo F.-S., Day B. M., Chen Y.-C., Tong M.-L., Mansikkamäki A., Layfield R. A. (2018). Science.

[cit4] Gould C. A., McClain K. R., Reta D., Kragskow J. G. C., Marchiori D. A., Lachman E., Choi E.-S., Analytis J. G., Britt R. D., Chilton N. F., Harvey B. G., Long J. R. (2022). Science.

[cit5] Lunghi A., Totti F., Sessoli R., Sanvito S. (2017). Nat. Commun..

[cit6] Escalera-Moreno L., Baldoví J. J., Gaita-Ariño A., Coronado E. (2018). Chem. Sci..

[cit7] Lunghi A., Sanvito S. (2019). Sci. Adv..

[cit8] Briganti M., Santanni F., Tesi L., Totti F., Sessoli R., Lunghi A. (2021). J. Am. Chem. Soc..

[cit9] Lunghi A. (2022). Sci. Adv..

[cit10] Kragskow J. G. C., Mattioni A., Staab J. K., Reta D., Skelton J. M., Chilton N. F. (2023). Chem. Soc. Rev..

[cit11] Nabi R., Staab J. K., Mattioni A., Kragskow J. G. C., Reta D., Skelton J. M., Chilton N. F. (2023). J. Am. Chem. Soc..

[cit12] Mariano L. A., Mondal S., Lunghi A. (2024). J. Chem. Theory Comput..

[cit13] Woodruff D. N., Winpenny R. E. P., Layfield R. A. (2013). Chem. Rev..

[cit14] tau, angmom_suite, spin_phonon_suite https://gitlab.com/chilton-group/

[cit15] Staab J. K., Rahman Md. K., Chilton N. F. (2024). Phys. Chem. Chem. Phys..

[cit16] Goodwin C. A. P., Ortu F., Reta D., Chilton N. F., Mills D. P. (2017). Nature.

[cit17] Parmar V. S., Thiel A. M., Nabi R., Gransbury G. K., Norre M. S., Evans P., Corner S. C., Skelton J. M., Chilton N. F., Mills D. P., Overgaard J. (2023). Chem. Commun..

[cit18] Togo A., Tanaka I. (2015). Scr. Mater..

[cit19] Staab J. K., Chilton N. F. (2022). J. Chem. Theory Comput..

[cit20] Garlatti E., Tesi L., Lunghi A., Atzori M., Voneshen D. J., Santini P., Sanvito S., Guidi T., Sessoli R., Carretta S. (2020). Nat. Commun..

[cit21] Togo A., Chaput L., Tanaka I. (2015). Phys. Rev. B: Condens. Matter Mater. Phys..

[cit22] Albino A., Benci S., Atzori M., Chelazzi L., Ciattini S., Taschin A., Bartolini P., Lunghi A., Righini R., Torre R., Totti F., Sessoli R. (2021). J. Phys. Chem. C.

[cit23] Chiesa A., Cugini F., Hussain R., Macaluso E., Allodi G., Garlatti E., Giansiracusa M., Goodwin C. A. P., Ortu F., Reta D., Skelton J. M., Guidi T., Santini P., Solzi M., De Renzi R., Mills D. P., Chilton N. F., Carretta S. (2020). Phys. Rev. B.

