# Supplementary material for: The impact of low-energy phonon lifetimes on the magnetic relaxation in a dysprosocenium single-molecule magnet
Source: Chem Commun (Camb). 2024 Oct 23;60(94):13915–8. doi: 10.1039/d4cc03768e (PMC11563352; doi:10.1039/d4cc03768e)
Supplement: CC-060-D4CC03768E-s001 [file CC-060-D4CC03768E-s001.pdf]

Supplementary Information (SI) for Chemical Communications.
This journal is © The Royal Society of Chemistry 2024

The impact of low-energy phonon lifetimes on the magnetic relaxation in a dysprosocenium single-molecule magnet

Rizwan Nabi,[a,#] Benjamin E. Atkinson,[b,#] Jakob K. Staab,[a] Jonathan M. Skelton[a,*] and Nicholas F. Chilton[a,b,*]

[a] Department of Chemistry, The University of Manchester, Manchester, M13 9PL, UK

[b] Research School of Chemistry, The Australian National University, Canberra, ACT, 2601, Australia

[#] These authors contributed equally to this work.

Email: jonathan.skelton@manchester.ac.uk, nicholas.chilton@anu.edu.au

Methods

We used density-functional theory (DFT) with VASP 5.4.4[1–4] to obtain an optimised crystal structure and evaluate the solid-state phonon modes, using the PBE functional[5] with the semi-empirical DFT-D3 dispersion correction[6] and projector augmented-wave (PAW) pseudopotentials including a 4f-in-core potential for Dy.[7,8] We used a plane-wave basis set with an energy cutoff of 850 eV and sampled the electronic structure at the Γ-point, justified by convergence of single-point energy and stress calculations. Starting from the primitive cell of **1** obtained from the Cambridge Structural Database (CCDC 2046705),[9] the atomic positions and unit cell parameters were optimised, maintaining the experimental unit cell volume, to total energy and force tolerances of 10^{-8} eV and 0.01 eV $Å^{-1}$. Phonons were calculated with Phonopy[10] from force constants evaluated in a 2×2×1 supercell. Third-order force constants were then calculated in the primitive cell using Phono3py.[11] Including all pairwise atomic displacements would require more than 2.7 million single-point calculations, so we employed various distance cutoffs of $3 < r_{\mathrm{cut}} < 7$ Å, in steps of 0.5 Å beyond which the pair-wise third-order force were assumed to be zero. This reduces the number of single-point computations to between 63,468 ($r_{\mathrm{cut}} = 3$ Å) and 674,244 ($r_{\mathrm{cut}} = 7$ Å). Linewidths were calculated using second-order perturbation theory on q-point grids with up to 5×5×5 subdivisions.

We used our established protocol for calculating the molecular spin-phonon coupling in the crystalline phase.[12–14] In this approach, we perform molecular state-average complete active space self-consistent field spin-orbit (SA-CASSCF-SO) calculations on a single cation of **1** surrounded by a spherical cluster of unit cells (approximate radius 30 Å, chosen by convergence testing). Molecules in the vicinity of the reference molecule are represented by point charges determined from gas-phase DFT calculations on each unique molecule, using Gaussian 09d,[15] with Dy substituted with Y to avoid a multiconfigurational ground state, and fitting the external molecular electrostatic potential using the CHELPG method.[16] The cluster is then embedded in a spherical conductor (Kirkwood solvent model with $\varepsilon \rightarrow \infty$) to screen the unphysical surface charges to accurately reproduce the long-range Madelung potential.[14]

Our SA-CASSCF-SO calculations are performed using OpenMolcas 23.02,[17] considering 18 $S = 5/2$ states (^{6}H and ^{6}F terms) in a 9-in-7 active space (4f^9 configuration) using the second-order Douglas-Kroll-Hess relativistic decoupling,[18] the Cholesky "atomic compact" resolution of the identity method for approximation of two electron integrals,[19] and ANO-RCC basis sets for all atoms (VTZP for Dy, VDZP for the first coordination sphere and VDZ for all other atoms).[20,21] These 18 spin-free states are then mixed with SO coupling, and the electronic states

corresponding to the $^6H_{15/2}$ multiplet are projected out of the *ab initio* basis to define the equilibrium Hamiltonian $\hat{H}$ (note that this is not just the lowest 16 Kramers doublets, but the entire angular momentum content of the multiplet). The spin-phonon coupling Hamiltonian for each phonon mode (index *j*) at each q-point, $\frac{\partial \hat{H}}{\partial Z_{\boldsymbol{q}j}}$, is evaluated using our linear vibronic coupling method,[14,22,23] and the components relevant to the $^6H_{15/2}$ multiplet are projected directly without recourse to a model Hamiltonian (see Figure S1 below for comparison of this approach to that using a model crystal field Hamiltonian). We note that our method does not include derivatives of the SO coupling operators, which are approximated using the atomic mean-field method;[24] recent work by Lunghi and co-workers has shown that this might have an non-zero effect on rates.[25]

Magnetic relaxation rates were determined using *TAU*,[12,13] considering both Orbach and Raman-I rate expressions, derived from perturbation theory, given by Equations 1–6 below.[13] Calculation of the rates using these perturbative expressions requires an energy gap within the lowest Kramers doublet, which occurs in the experiment due to the presence of a dipolar magnetic field and/or the driving AC magnetic field, and thus we apply a small magnetic field of 2 Oe along the main magnetic axis of the molecule to split the ground doublet by *ca.* 0.02 cm^{-1}. The Raman-I integral is restricted to $\omega < 99$ cm^{-1} to avoid divergence in the perturbation expression, which is sufficiently smaller than the first crystal field excitation of 457.47 cm^{-1} while including sufficient modes populated in the Raman region < 60 K (Figure S2). Integration is performed over anti-Lorentzian phonon lineshapes (Equation 9 below)[13] to an equivalent range of $\mu \pm 2\sigma$ (95%) using the trapezoidal method with 100 equidistant steps.

$$\gamma_{\mathrm{fi}}^{-} = \frac{2\pi}{\hbar}\sum_{\boldsymbol{q}j}\int \left|\left\langle \psi_{\mathrm{f}}\middle|\hat{V}_{\boldsymbol{q}j}^{(1e)}\middle|\psi_{\mathrm{i}}\right\rangle\right|^2 \bar{n}(\hbar\omega)\delta(E_{\mathrm{f}} - E_{\mathrm{i}} - \hbar\omega)\rho_{\boldsymbol{q}j}(\hbar\omega)\mathrm{d}\hbar\omega$$

Equation 1

$$\gamma_{\mathrm{fi}}^{+} = \frac{2\pi}{\hbar}\sum_{\boldsymbol{q}j}\int \left|\left\langle \psi_{\mathrm{f}}\middle|\hat{V}_{\boldsymbol{q}j}^{(1e)}\middle|\psi_{\mathrm{i}}\right\rangle\right|^2 (\bar{n}(\hbar\omega) + 1)\delta(E_{\mathrm{f}} - E_{\mathrm{i}} + \hbar\omega)\rho_{\boldsymbol{q}j}(\hbar\omega)\mathrm{d}\hbar\omega$$

Equation 2

$$\gamma_{\mathrm{fi}}{}^{\mathrm{I},--} = \frac{\pi}{\hbar} \sum_{\boldsymbol{q}j,\boldsymbol{q}'j'} \int\int \left| \sum_{\mathrm{c}} \frac{\left\langle \psi_{\mathrm{f}} \middle| \hat{V}^{(1e)}_{\boldsymbol{q}j} \middle| \psi_{\mathrm{c}} \right\rangle \left\langle \psi_{\mathrm{c}} \middle| \hat{V}^{(1e)}_{\boldsymbol{q}'j'} \middle| \psi_{\mathrm{i}} \right\rangle}{E_{\mathrm{c}} - E_{\mathrm{i}} - \hbar\omega'} + \frac{\left\langle \psi_{\mathrm{f}} \middle| \hat{V}^{(1e)}_{\boldsymbol{q}'j'} \middle| \psi_{\mathrm{c}} \right\rangle \left\langle \psi_{\mathrm{c}} \middle| \hat{V}^{(1e)}_{\boldsymbol{q}j} \middle| \psi_{\mathrm{i}} \right\rangle}{E_{\mathrm{c}} - E_{\mathrm{i}} - \hbar\omega} \right|^2 \times \bar{n}(\hbar\omega)\bar{n}(\hbar\omega')\delta(E_{\mathrm{f}} - E_{\mathrm{i}} - \hbar\omega - \hbar\omega')\rho_{\boldsymbol{q}j}(\hbar\omega)\rho_{\boldsymbol{q}'j'}(\hbar\omega')\mathrm{d}\hbar\omega\mathrm{d}\hbar\omega'$$

Equation 3

$$\gamma_{\mathrm{fi}}{}^{\mathrm{I},++} = \frac{\pi}{\hbar} \sum_{\boldsymbol{q}j,\boldsymbol{q}'j'} \int\int \left| \sum_{\mathrm{c}} \frac{\left\langle \psi_{\mathrm{f}} \middle| \hat{V}^{(1e)}_{\boldsymbol{q}j} \middle| \psi_{\mathrm{c}} \right\rangle \left\langle \psi_{\mathrm{c}} \middle| \hat{V}^{(1e)}_{\boldsymbol{q}'j'} \middle| \psi_{\mathrm{i}} \right\rangle}{E_{\mathrm{c}} - E_{\mathrm{i}} + \hbar\omega'} + \frac{\left\langle \psi_{\mathrm{f}} \middle| \hat{V}^{(1e)}_{\boldsymbol{q}'j'} \middle| \psi_{\mathrm{c}} \right\rangle \left\langle \psi_{\mathrm{c}} \middle| \hat{V}^{(1e)}_{\boldsymbol{q}j} \middle| \psi_{\mathrm{i}} \right\rangle}{E_{\mathrm{c}} - E_{\mathrm{i}} + \hbar\omega} \right|^2 \times (\bar{n}(\hbar\omega) + 1)(\bar{n}(\hbar\omega') + 1)\delta(E_{\mathrm{f}} - E_{\mathrm{i}} + \hbar\omega + \hbar\omega')\rho_{\boldsymbol{q}j}(\hbar\omega)\rho_{\boldsymbol{q}'j'}(\hbar\omega')\mathrm{d}\hbar\omega\mathrm{d}\hbar\omega'$$

Equation 4

$$\gamma_{\mathrm{fi}}{}^{\mathrm{I},-+} = \frac{\pi}{\hbar} \sum_{\boldsymbol{q}j,\boldsymbol{q}'j'} \int\int \left| \sum_{\mathrm{c}} \frac{\left\langle \psi_{\mathrm{f}} \middle| \hat{V}^{(1e)}_{\boldsymbol{q}j} \middle| \psi_{\mathrm{c}} \right\rangle \left\langle \psi_{\mathrm{c}} \middle| \hat{V}^{(1e)}_{\boldsymbol{q}'j'} \middle| \psi_{\mathrm{i}} \right\rangle}{E_{\mathrm{c}} - E_{\mathrm{i}} + \hbar\omega'} + \frac{\left\langle \psi_{\mathrm{f}} \middle| \hat{V}^{(1e)}_{\boldsymbol{q}'j'} \middle| \psi_{\mathrm{c}} \right\rangle \left\langle \psi_{\mathrm{c}} \middle| \hat{V}^{(1e)}_{\boldsymbol{q}j} \middle| \psi_{\mathrm{i}} \right\rangle}{E_{\mathrm{c}} - E_{\mathrm{i}} - \hbar\omega} \right|^2 \times \bar{n}(\hbar\omega)(\bar{n}(\hbar\omega') + 1)\delta(E_{\mathrm{f}} - E_{\mathrm{i}} - \hbar\omega + \hbar\omega')\rho_{\boldsymbol{q}j}(\hbar\omega)\rho_{\boldsymbol{q}'j'}(\hbar\omega')\mathrm{d}\hbar\omega\mathrm{d}\hbar\omega'$$

Equation 5

$$\gamma_{\mathrm{fi}}{}^{\mathrm{I},+-} = \frac{\pi}{\hbar} \sum_{\boldsymbol{q}j,\boldsymbol{q}'j'} \int\int \left| \sum_{\mathrm{c}} \frac{\left\langle \psi_{\mathrm{f}} \middle| \hat{V}_{\boldsymbol{q}j}^{(1e)} \middle| \psi_{\mathrm{c}} \right\rangle \left\langle \psi_{\mathrm{c}} \middle| \hat{V}_{\boldsymbol{q}'j'}^{(1e)} \middle| \psi_{\mathrm{i}} \right\rangle}{E_{\mathrm{c}} - E_{\mathrm{i}} - \hbar\omega'} + \frac{\left\langle \psi_{\mathrm{f}} \middle| \hat{V}_{\boldsymbol{q}'j'}^{(1e)} \middle| \psi_{\mathrm{c}} \right\rangle \left\langle \psi_{\mathrm{c}} \middle| \hat{V}_{\boldsymbol{q}j}^{(1e)} \middle| \psi_{\mathrm{i}} \right\rangle}{E_{\mathrm{c}} - E_{\mathrm{i}} + \hbar\omega} \right|^2$$
$$\times \left(\bar{n}(\hbar\omega) + 1\right)\bar{n}(\hbar\omega')\delta(E_{\mathrm{f}} - E_{\mathrm{i}} + \hbar\omega - \hbar\omega')\rho_{\boldsymbol{q}j}(\hbar\omega)\rho_{\boldsymbol{q}'j'}(\hbar\omega')\mathrm{d}\hbar\omega\mathrm{d}\hbar\omega'$$

Equation 6

$$\bar{n}(\hbar\omega) = \frac{1}{\exp(\hbar\omega/k_{\mathrm{B}}T) - 1}$$

Equation 7

$$L_{\boldsymbol{q}j}^{\pm}(\hbar\omega) = \frac{\Gamma_{\boldsymbol{q}j}}{2\pi\left[\left(\Gamma_{\boldsymbol{q}j}/2\right)^2 + \left(\hbar\omega \pm \hbar\omega_{\boldsymbol{q}j}\right)^2\right]}$$

Equation 8

$$\rho_{\boldsymbol{q}j}(\hbar\omega) = \pi\left[\frac{L_{\boldsymbol{q}j}^{-}(\hbar\omega) - L_{\boldsymbol{q}j}^{+}(\hbar\omega)}{2\tan^{-1}\left(2\hbar\omega_{\boldsymbol{q}j}/\Gamma_{\boldsymbol{q}j}\right)}\right]$$

Equation 9

Comparison of the model-free rates (used for all of the work herein) and those obtained by considering the dynamics using a model Hamiltonian parameterised by a crystal-field approach shows that the two methods give very similar results, differing by an average factor of 2 in the Orbach region and an average factor of 4 in the Raman-I region (Figure S1).

a) Raman **b)** Orbach **c)** Total

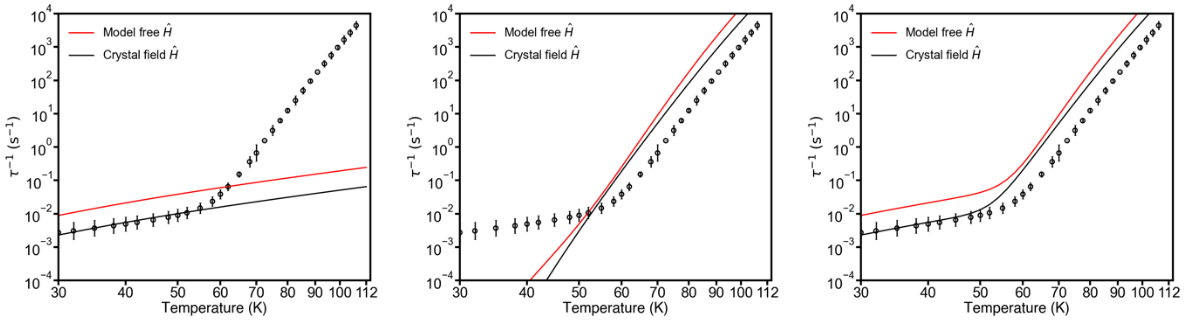

Figure S1. Experimental (black circles) and calculated a) Raman, b) Orbach and c) total magnetic reversal rates for **1** as a function of temperature with fixed phonon linewidth of Γ = 10 cm^{-1}, employing direct Hamiltonian derivatives (red) and crystal field model derivatives (black). The calculations were performed using the solid-state phonon modes evaluated on a 1×1×1 *q*-point mesh. Error bars on the experimental rates correspond to one estimated standard deviation.[26,27]

Table S1. Optimised lattice parameters of the primitive unit cell of **1** compared to experimental measurements.[9]

Parameter	Experimental	Optimised
**a (Å)**	11.6278(3)	11.63269
**b (Å)**	12.6173(5)	12.59458
**c (Å)**	20.3029(7)	20.34757
α **(°)**	107.031(3)	107.0157
β **(°)**	96.387(3)	96.7115
γ **(°)**	99.377(3)	99.3399

Table S2. Electronic structure of **1** calculated with the crystal field parameters obtained from CASSCF-SO at the DFT optimised geometry. Each row corresponds to a Kramers doublet.

Energy (cm^{-1})	Energy (K)	g_x	g_y	g_z	Angle[a] (deg)	Wavefunction[b]	$<J_z>$
0.00	0.00	0.00	0.00	19.99	--	99.7% |± 15/2⟩	7.49
465.28	664.78	0.00	0.00	17.09	2.26	99.7% |± 13/2⟩	6.50
713.58	1019.55	0.00	0.00	14.42	4.31	97.5% |± 11/2⟩ + 1.1% |± 7/2⟩ + 0.8% |± 9/2⟩	5.48
883.82	1262.79	0.03	0.04	11.74	4.40	96.9% |± 9/2⟩ + 2.1% |± 5/2⟩ + 0.8% |± 11/2⟩	4.46
1043.52	1490.98	0.17	0.25	9.10	4.70	97.0% |± 7/2⟩ + 1.6% |± 3/2⟩ + 1.0% |± 11/2⟩	3.49
1192.13	1703.30	1.12	1.63	6.47	2.79	96.5% |± 5/2⟩ + 2.1% |± 9/2⟩ + 0.8% |± 1/2⟩	2.51
1307.27	1867.81	0.89	1.93	3.87	1.13	97.2% |± 3/2⟩ + 1.6% |± 7/2⟩ + 0.8% |∓ 1/2⟩	1.51
1369.55	1956.80	1.30	9.97	11.13	89.88	98.1% |± 1/2⟩ + 0.9% |± 5/2⟩ + 0.7% |∓ 3/2⟩	0.50

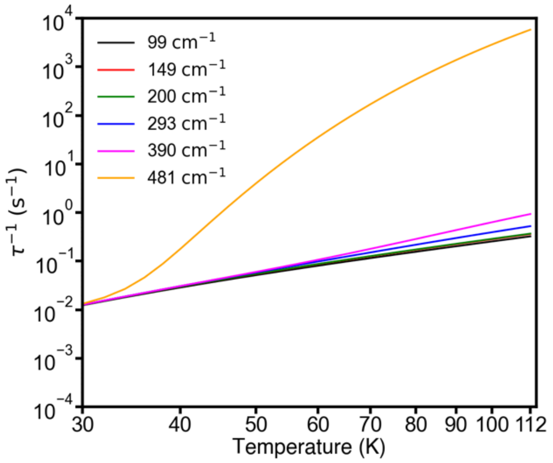

Figure S2. Calculated Raman total magnetic reversal rates for **1** as a function of temperature obtained with solid-state phonon modes, evaluated including phonons with frequencies up to 99 (black), 149 (red), 200 (green), 293 (blue), 390 (pink) and 481 cm^{-1}. Evaluated on a 3×3×3 q-point mesh with a fixed phonon linewidth Γ = 1 cm.

a) Raman **b)** Orbach **c)** Total

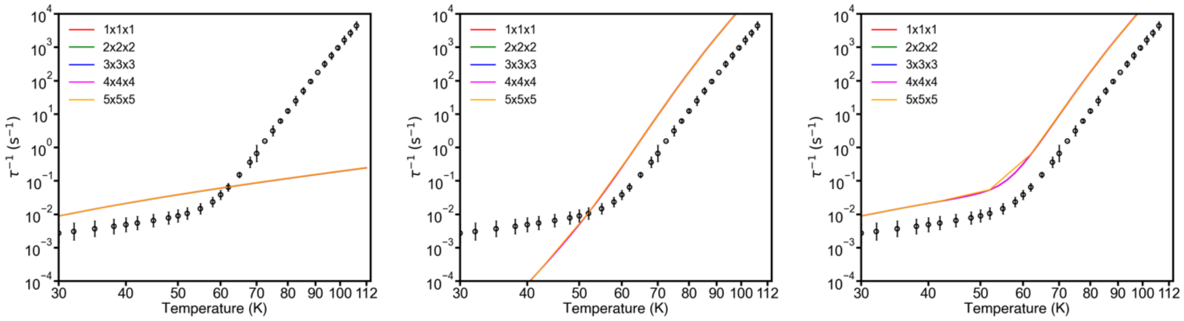

Figure S3. Experimental (black circles) and calculated a) Raman, b) Orbach and c) total magnetic reversal rates for **1** as a function of temperature obtained with solid-state phonon modes evaluated on 1×1×1 (red), 2×2×2 (green), 3×3×3 (blue), 4×4×4 (magenta) and 5×5×5 (orange) *q*-point meshes. Imaginary modes were excluded from the calculations, and a fixed phonon linewidth $\Gamma = 10$ cm^{-1} was used. Error bars on the experimental rates correspond to one estimated standard deviation.[26,27]

a) Raman **b)** Orbach **c)** Total

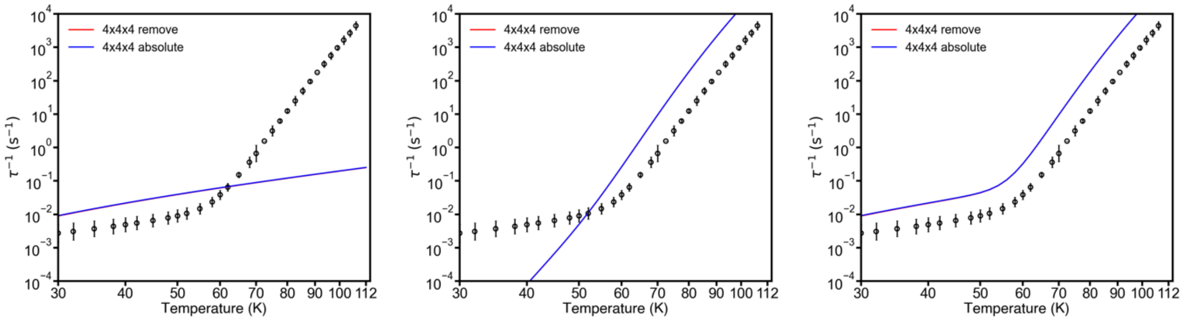

Figure S4. Experimental (black circles) and calculated a) Raman, b) Orbach and c) total magnetic reversal rates for **1** as a function of temperature obtained with imaginary modes excluded from the calculations (red) or with their frequencies set to the absolute values (blue). The calculations were performed with the solid-state phonon modes evaluated on a 4×4×4 *q*-point mesh and a fixed phonon linewidth $\Gamma = 10$ cm^{-1}. Error bars on the experimental rates correspond to one estimated standard deviation.[26,27]

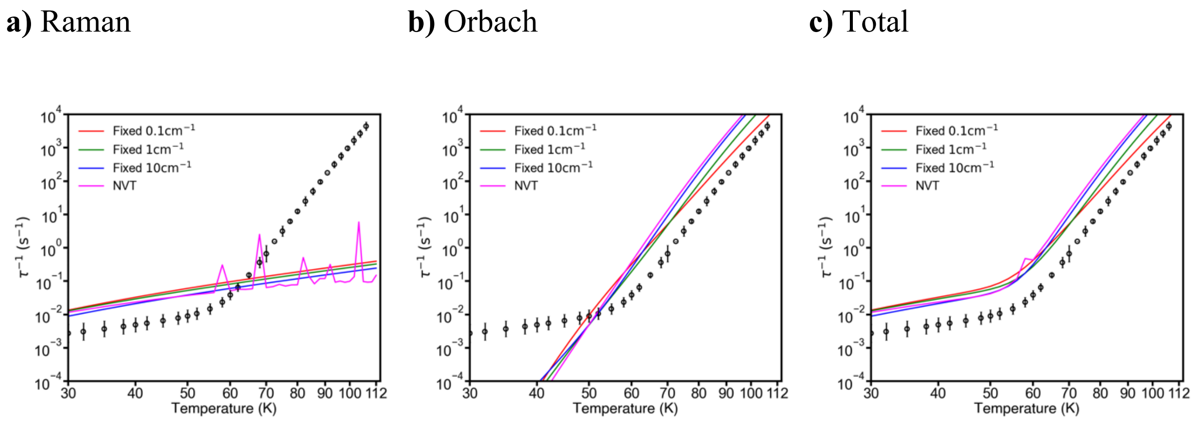

Figure S5. Experimental (black circles) and calculated a) Raman, b) Orbach and c) total magnetic reversal rates for **1** as a function of temperature obtained with fixed phonon linewidths of Γ = 0.1 (red), 1 (green) and 10 cm^{-1} (blue), and with linewidths from the NVT approximation[28] (magenta). The calculations were performed with solid-state phonon modes evaluated on a 1×1×1 q-point mesh. Error bars on the experimental rates correspond to one estimated standard deviation.[26,27] The total NVT rates exclude the Raman contribution at $T \geq$ 62 K due to numerical instabilities.

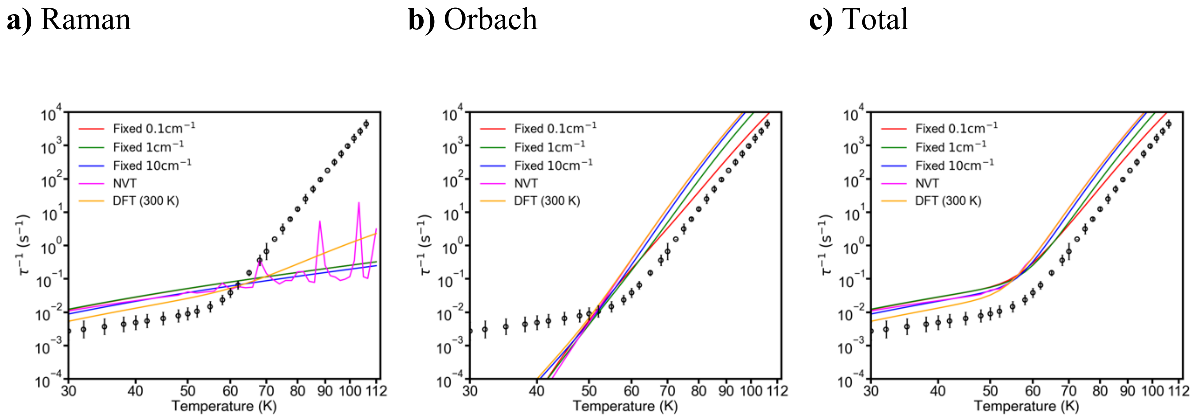

Figure S6. Experimental (black circles) and calculated a) Raman, b) Orbach and c) total magnetic reversal rates for **1** as a function of temperature obtained with fixed phonon linewidths of Γ = 0.1 (red), 1 (green) and 10 cm^{-1} (blue), with linewidths from the NVT approximation[28] (magenta), and with DFT linewidths calculated at 300 K (orange). The calculations were performed with solid-state phonon modes evaluated on a 2×2×2 q-point mesh. Error bars on the experimental rates correspond to one estimated standard deviation.[26,27] The total NVT rates exclude the Raman contribution at $T \geq$ 62 K due to numerical instabilities.

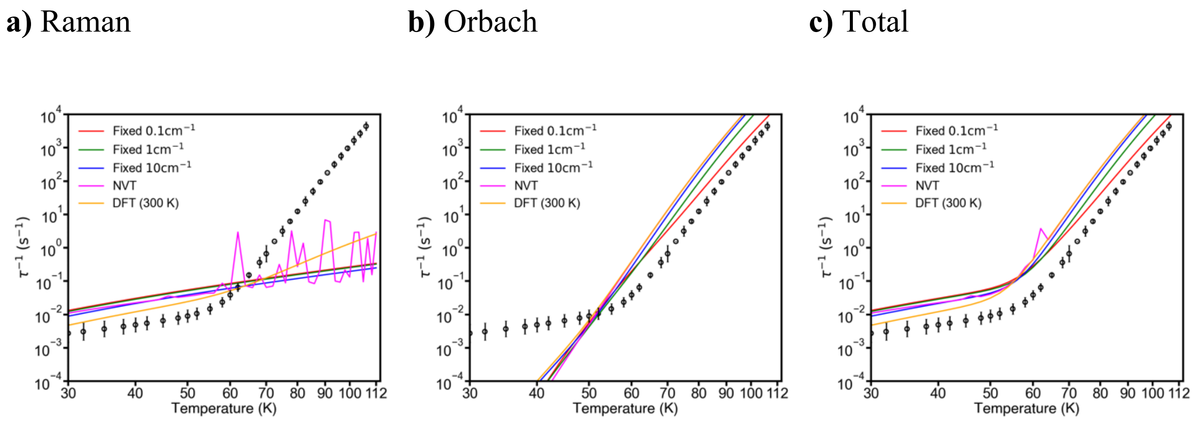

Figure S7. Experimental (black circles) and calculated a) Raman, b) Orbach and c) total magnetic reversal rates for **1** as a function of temperature obtained with fixed phonon linewidths of Γ = 0.1 (red), 1 (green) and 10 (blue) cm^{-1}, with linewidths from the NVT approximation[28] (magenta), and with DFT linewidths calculated at 300 K (orange). The calculations were performed with solid-state phonon modes evaluated on a 3×3×3 *q*-point mesh. Error bars on the experimental rates correspond to one estimated standard deviation.[26,27] The total NVT rates exclude the Raman contribution at $T \geq 62$ K due to numerical instabilities.

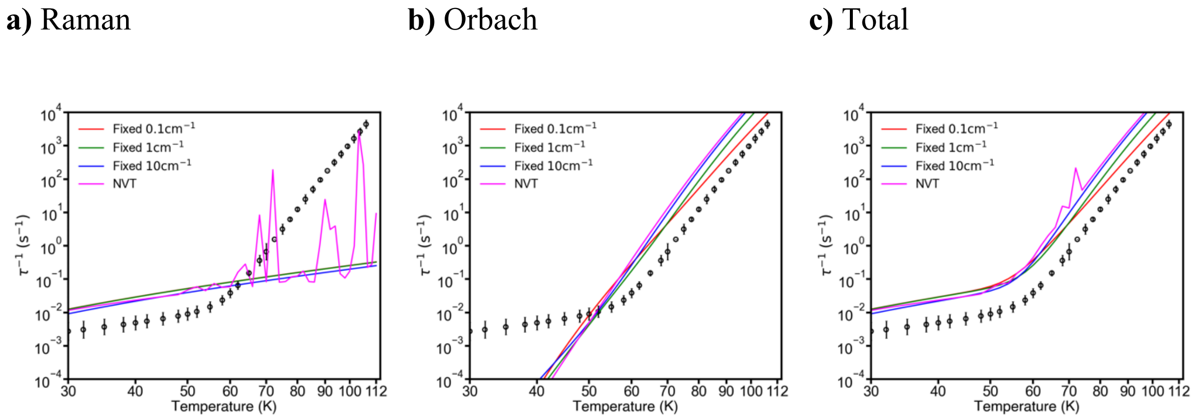

Figure S8. Experimental (black circles) and calculated a) Raman, b) Orbach and c) total magnetic reversal rates for **1** as a function of temperature obtained with fixed phonon linewidths of Γ = 0.1 (red), 1 (green) and 10 (blue) cm^{-1}, and with linewidths from the NVT approximation[28] (magenta). The calculations were performed using the solid-state phonon modes evaluated on a 4×4×4 *q*-point mesh, and the frequencies of imaginary modes were set to their absolute values. Error bars on the experimental rates correspond to one estimated standard deviation.[26,27] The total NVT rates exclude the Raman contribution at $T \geq 62$ K due to numerical instabilities.

a) Raman **b)** Orbach **c)** Total

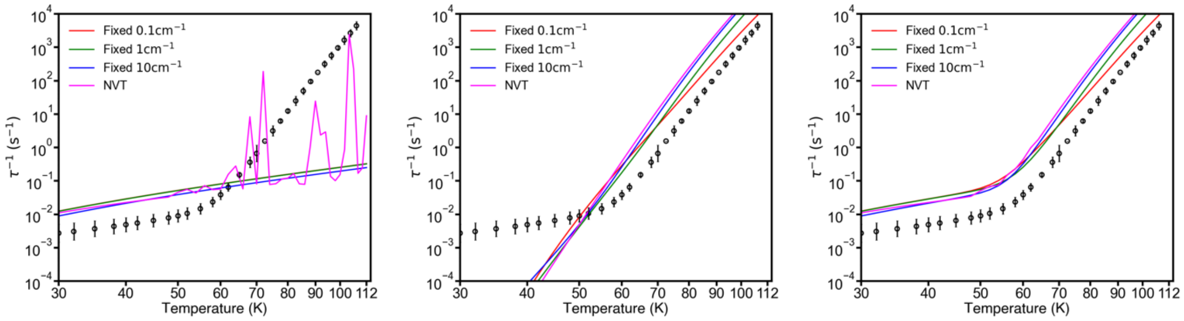

Figure S9. Experimental (black circles) and calculated a) Raman, b) Orbach and c) total magnetic reversal rates for **1** as a function of temperature obtained with fixed phonon linewidths of Γ = 0.1 (red), 1 (green) and 10 (blue) cm^{-1}, and with linewidths from the NVT approximation[28] (magenta). The calculations were performed using the solid-state phonon modes evaluated on a 4×4×4 q-point mesh, and imaginary modes were excluded. Error bars on the experimental rates correspond to one estimated standard deviation.[26,27] The total NVT rates exclude the Raman contribution at $T \geq 62$ K due to numerical instabilities.

a) Raman **b)** Orbach **c)** Total

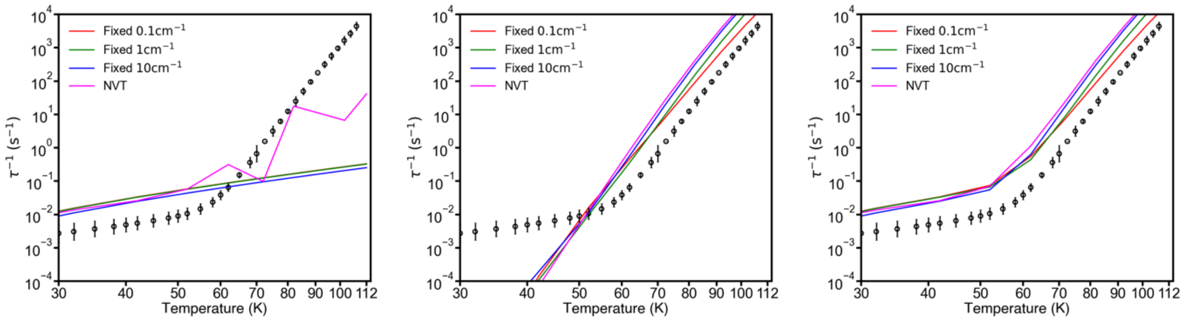

Figure S10. Experimental (black circles) and calculated a) Raman, b) Orbach and c) total magnetic reversal rates for **1** as a function of temperature with fixed phonon linewidths of Γ = 0.1 (red), 1 (green) and 10 (blue) cm^{-1}, and with linewidths from the NVT approximation[28] (magenta). The calculations were performed using the solid-state phonon modes evaluated on a 5×5×5 q-point mesh, and the frequencies of imaginary modes were set to their absolute values. Error bars on the experimental rates correspond to one estimated standard deviation.[26,27] The total NVT rates exclude the Raman contribution at $T \geq 62$ K due to numerical instabilities.

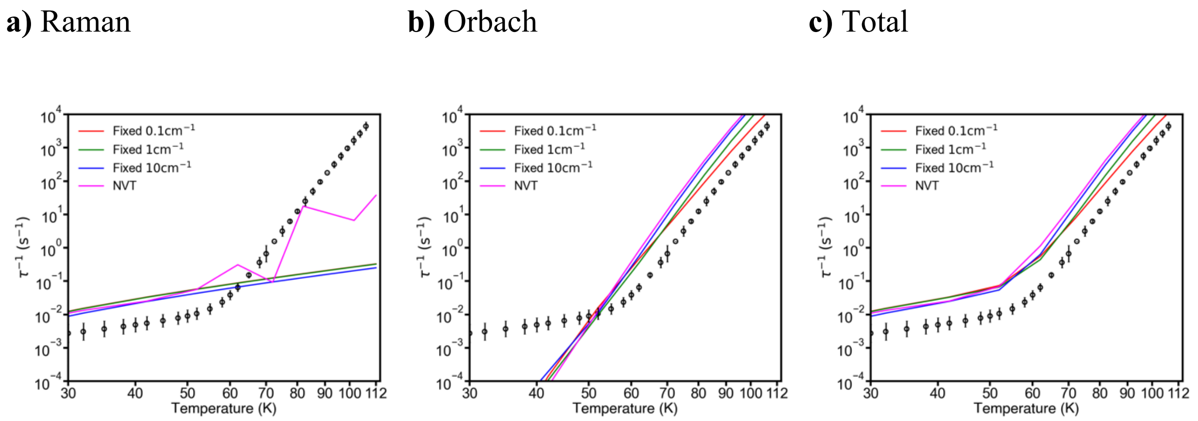

Figure S11. Experimental (black circles) and calculated a) Raman, b) Orbach and c) total magnetic reversal rates for **1** as a function of temperature with fixed phonon linewidths of Γ = 0.1 (red), 1 (green) and 10 (blue) cm^{-1}, and with linewidths from the NVT approximation[28] (magenta). The calculations were performed using the solid-state phonon modes evaluated on a 5×5×5 q-point mesh, and imaginary modes were excluded. Error bars on the experimental rates correspond to one estimated standard deviation.[26,27] The total NVT rates exclude the Raman contribution at $T \geq 62$ K due to numerical instabilities.

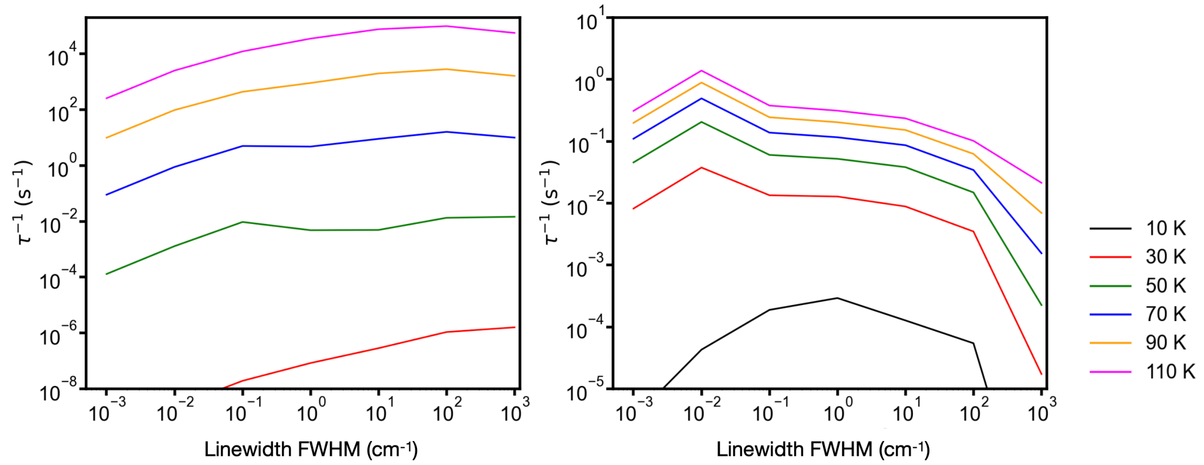

Figure S12. Magnetic reversal rates of **1** as a function of full-width half-maximum (FWHM) linewidth. Calculations are performed on the 1×1×1 q-point mesh at different fixed temperatures.

In our previous work we explained the decrease in Raman-I rates as a function of increasing linewidth as arising due to the Bose-Einstein occupation terms that diminish contributions from high-energy phonons at a finite temperature.[14] This occurs when large linewidths smear out phonon modes to higher and lower energies, but there is a hard limit that the pDOS must approach zero at zero energy, which is enforced in our method by the use of anti-Lorentzian lineshapes (Equation 9). We show the effect empirically in our rates for compound **1** above (Figure S12), but we sought to show in a more direct context how this arises. Considering one of the Raman-I terms (Equation 6), we simplify the expression by considering only one phonon mode $\boldsymbol{q}j = \boldsymbol{q}'j'$ from which both phonon energies $\hbar\omega$ and $\hbar\omega'$ are taken, in a system with only one excited state $|\psi_\mathrm{c}\rangle$, and further set $\left\langle\psi_\mathrm{f}\middle|\hat{V}_{\boldsymbol{q}j}^{(1e)}\middle|\psi_\mathrm{c}\right\rangle = \left\langle\psi_\mathrm{c}\middle|\hat{V}_{\boldsymbol{q}'j'}^{(1e)}\middle|\psi_\mathrm{i}\right\rangle = \left\langle\psi_\mathrm{f}\middle|\hat{V}_{\boldsymbol{q}'j'}^{(1e)}\middle|\psi_\mathrm{c}\right\rangle = \left\langle\psi_\mathrm{c}\middle|\hat{V}_{\boldsymbol{q}j}^{(1e)}\middle|\psi_\mathrm{i}\right\rangle = 1$, $\pi = 1$ and $\hbar = 1$, and define $E_\mathrm{f} = 0$ and $\hbar\omega' = \hbar\omega - E_\mathrm{i}$ to give:

$$\gamma = \int \left(\frac{1}{E_\mathrm{c} - \hbar\omega} + \frac{1}{E_\mathrm{c} - E_\mathrm{i} + \hbar\omega}\right)^2 (\bar{n}(\hbar\omega) + 1)\bar{n}(\hbar\omega - E_\mathrm{i})\rho_{\boldsymbol{q}j}(\hbar\omega)\rho_{\boldsymbol{q}j}(\hbar\omega - E_\mathrm{i})\mathrm{d}\hbar\omega$$

Equation 10

Considering the case of $E_\mathrm{c} \gg k_\mathrm{B}T$ and $E_\mathrm{i} \ll k_\mathrm{B}T$ (e.g. $E_\mathrm{c} = 500$ cm^{-1}, $E_\mathrm{i} = 0.02$ cm^{-1} and 10 K $< T <$ 100 K, valid for Dy(III) SMMs such as the present compound **1**), we perform numeric integrals of Equation 10 using Mathematica as a function of FWHM linewidth in the range 10^{-3} cm^{-1} $\leq \Gamma \leq 10^3$ cm^{-1} for a range of temperatures and central phonon energies $\hbar\omega_{\boldsymbol{q}j}$ (Figure S13). The results for $\hbar\omega_{\boldsymbol{q}j} = 10$ cm^{-1} and $\hbar\omega_{\boldsymbol{q}j} = 30$ cm^{-1} are qualitatively in agreement with the full calculation results (Figure S12), showing the same profiles and temperature dependence. In all but a few circumstances, the rate decreases with increasing linewidth, as we found empirically in our full calculations on compound **1** and in prior work.[14] The cases where there is a positive correlation between rates and linewidths are when: i) $\Gamma < E_\mathrm{i}$, and ii) when $k_\mathrm{B}T \ll \hbar\omega_{\boldsymbol{q}j}$ and $\Gamma \sim \hbar\omega_{\boldsymbol{q}j}$ (e.g. Figure S13e at 10 K for $\Gamma\sim$ 50 cm^{-1}). Case i) does not in the full calculations on compound **1** as $E_\mathrm{i} \sim 0.02$ cm^{-1} and $\Gamma > 0.1$ cm^{-1} for all modes (Figure 3a). The positive correlation in case ii) is far-outweighed by the negative correlation for lower energy modes at the same temperature: i.e., the positively-correlated rates for $\hbar\omega_{\boldsymbol{q}j} = 100$ cm^{-1} at 10 K and $\Gamma\sim$ 50 cm^{-1} (Figure S13e) are on the order of 10^{-11} a.u., while the rates for $\hbar\omega_{\boldsymbol{q}j} =$ 10 cm^{-1} at 10 K and $\Gamma\sim$ 50 cm^{-1} (Figure S13a) have a negative correlation and are on the order of 10^{-8} a.u. Thus, the latter case is also not observed when integrating all phonon modes in the low-energy region as we perform in our full calculations.

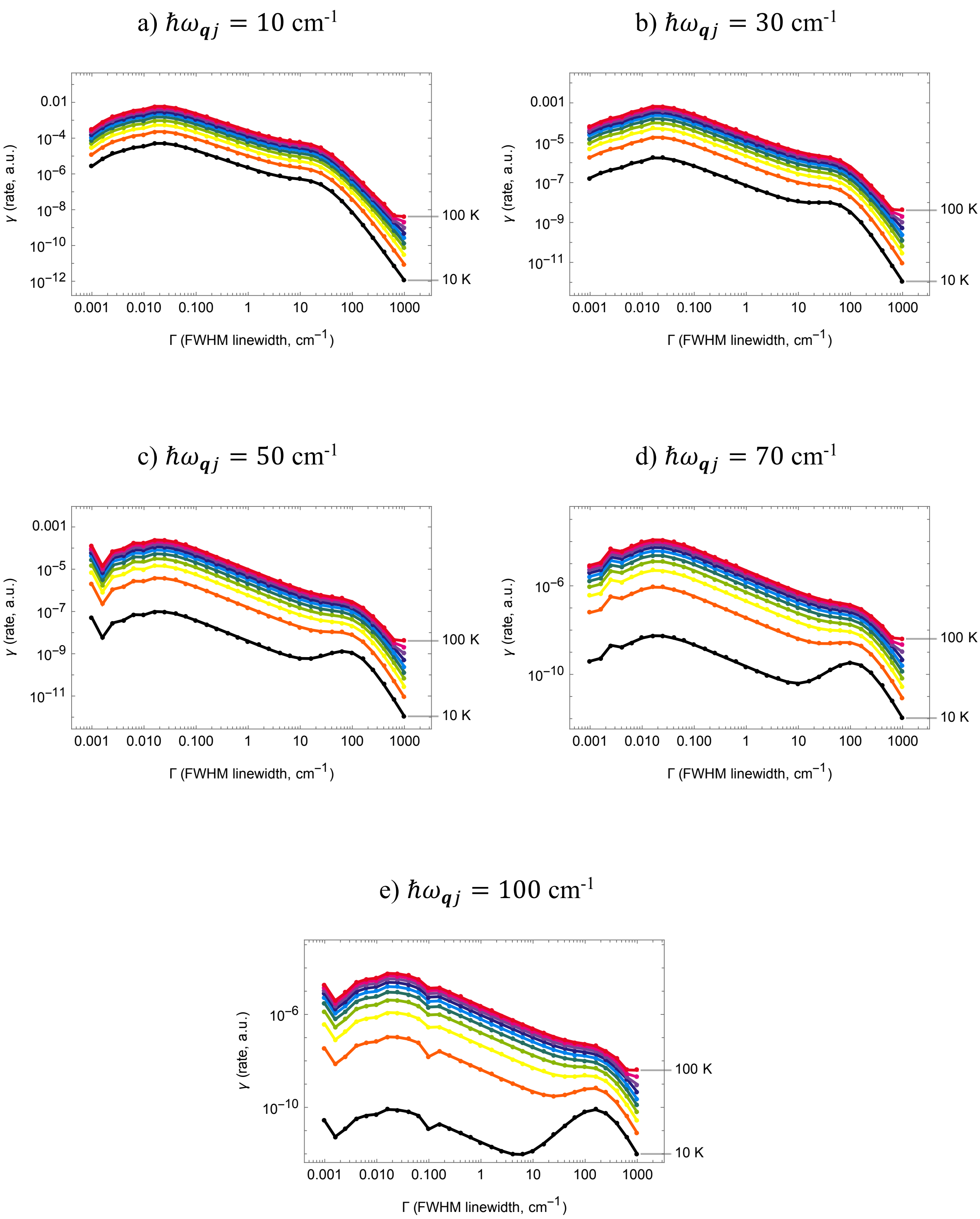

Figure S13. Model rates (Equation 10) as a function of linewidth at 10 (black), 20 (orange), 30 (yellow), 40 (light green), 50 (dark green), 60 (light blue), 70 (dark blue), 80 (purple), 90 (pink) and 100 K (red) for a selection of central phonon mode energies. Discontinuities in the $\hbar\omega_{qj}$ = 50 cm^{-1}, $\hbar\omega_{qj}$ = 70 cm^{-1} and $\hbar\omega_{qj}$ = 100 cm^{-1} data are due to numerical instabilities in the integration in Mathematica.

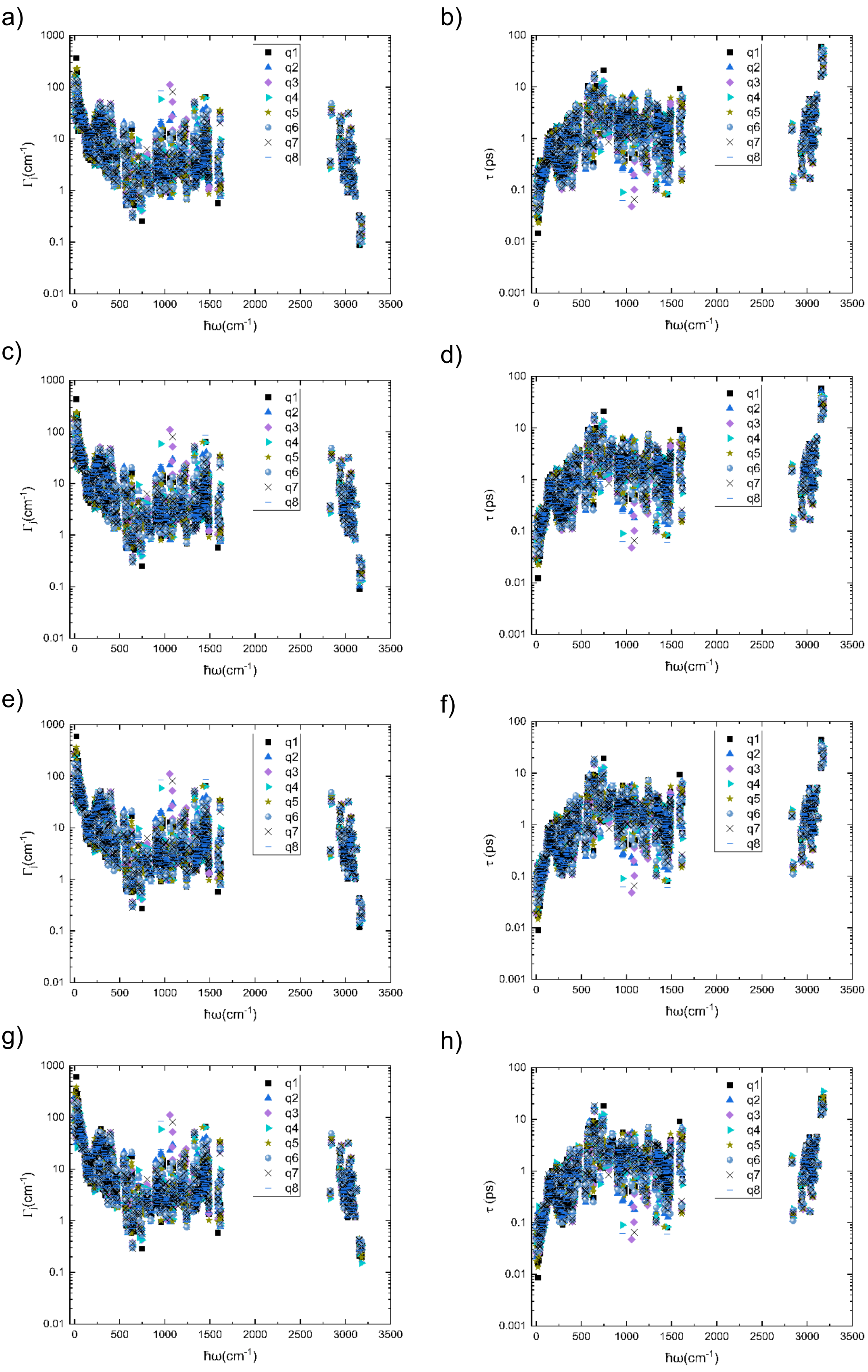

a)
b)
c)
d)
e)
f)
g)
h)
Γj(cm-1)
τ (ps)
ħω(cm-1)
q1
q2
q3
q4
q5
q6
q7
q8

i)

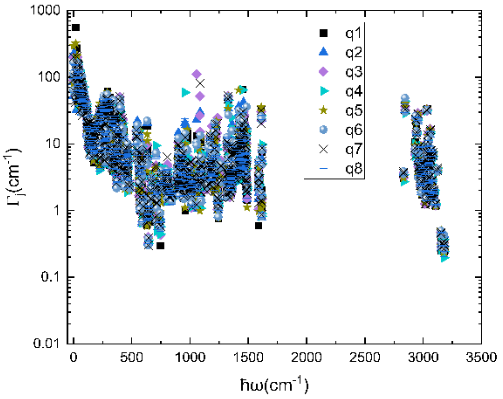

Γj(cm-1)
ħω(cm-1)
q1
q2
q3
q4
q5
q6
q7
q8

j)

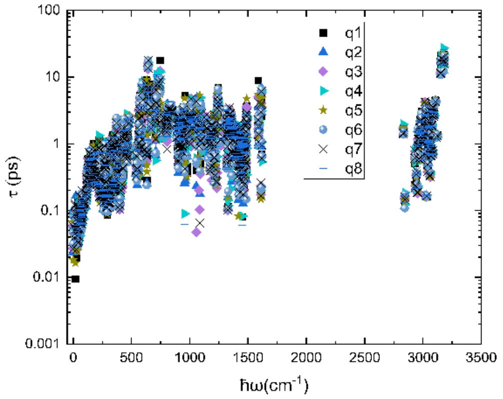

τ (ps)
ħω(cm-1)
q1
q2
q3
q4
q5
q6
q7
q8

k)

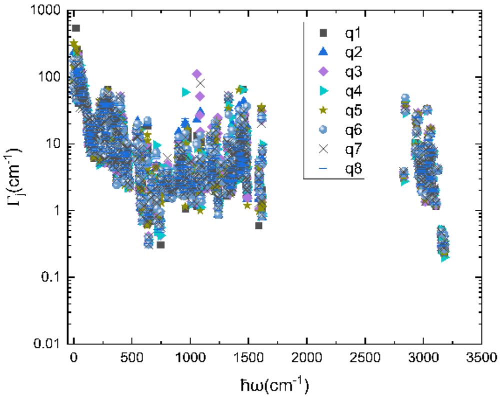

Γj(cm-1)
ħω(cm-1)
q1
q2
q3
q4
q5
q6
q7
q8

l)

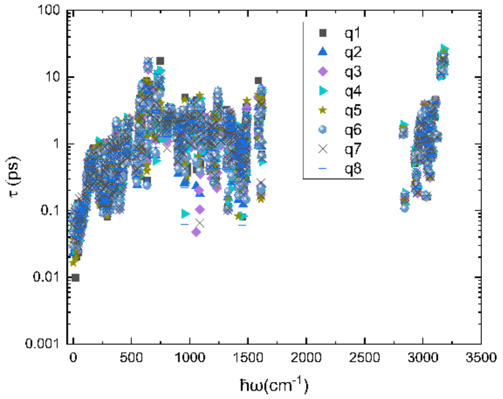

τ (ps)
ħω(cm-1)
q1
q2
q3
q4
q5
q6
q7
q8

m)

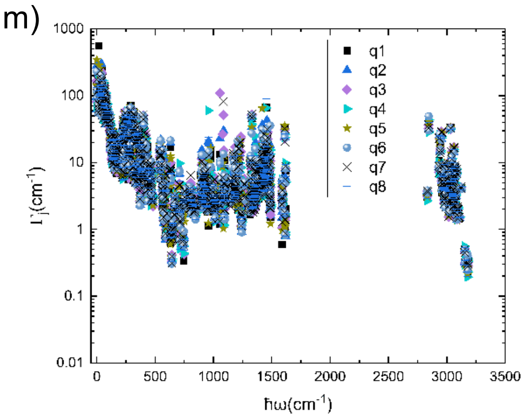

Γj(cm-1)
ħω(cm-1)
q1
q2
q3
q4
q5
q6
q7
q8

n)

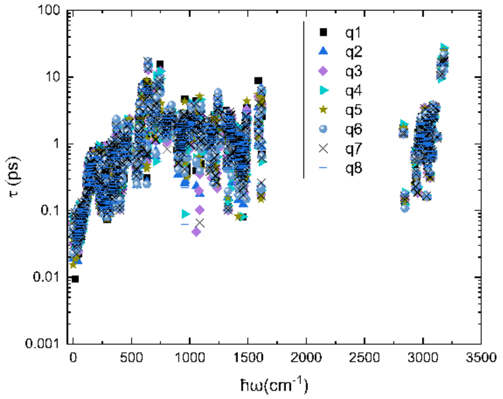

τ (ps)
ħω(cm-1)
q1
q2
q3
q4
q5
q6
q7
q8

o)

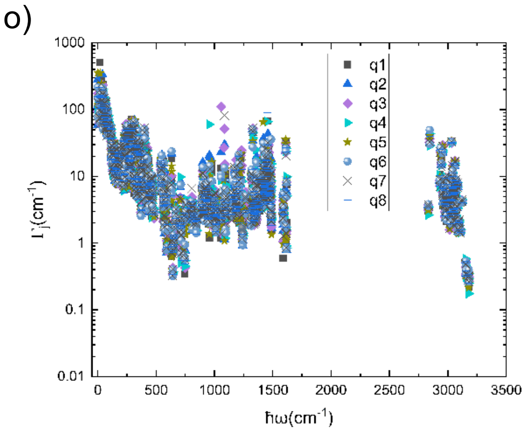

Γj(cm-1)
ħω(cm-1)
q1
q2
q3
q4
q5
q6
q7
q8

p)

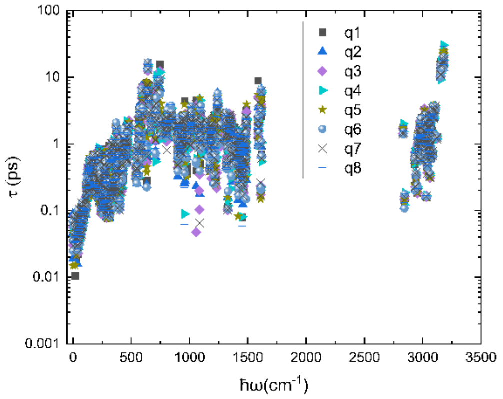

τ (ps)
ħω(cm-1)
q1
q2
q3
q4
q5
q6
q7
q8

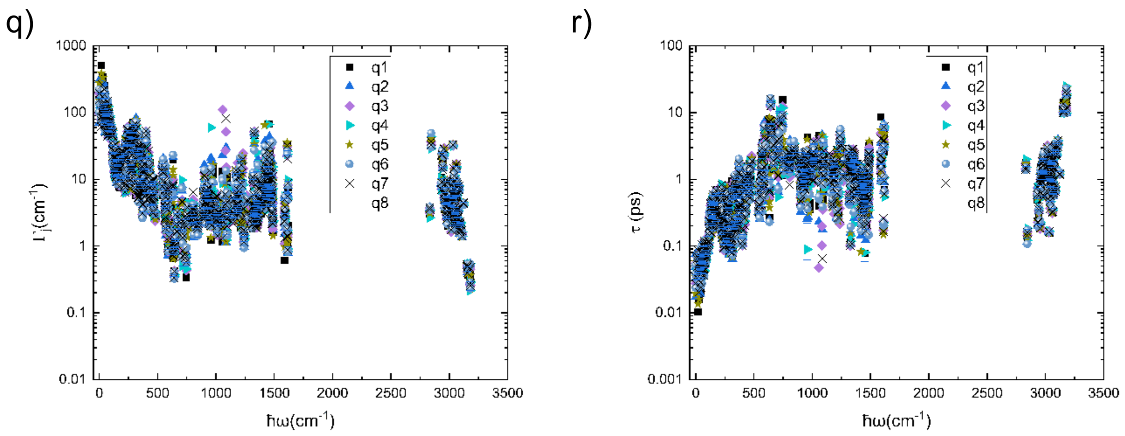

Figure S14. Calculated phonon linewidths (left) and phonon lifetimes (right) as a function of mode energy at 300 K, at the unique *q*-points in a 2×2×2 mesh, calculated with pair-distance cutoffs of r_{cut} = 3 (top row) to 7 Å (bottom row).

Table S3. Minimum, maximum, average and standard deviation (SD) of the differences between the calculated 300 K phonon lifetimes of **1**, evaluated on a 2×2×2 *q*-point mesh and obtained with pair-distance cutoffs of r_{cut} = 3-7 Å, compared to the largest r_{cut} = 7 Å.

	**Δ [ps]**				**Δ [%]**			
r_{cut} **[Å]**	**Min.**	**Max.**	**Avg.**	**SD**	**Min.**	**Max.**	**Avg.**	**SD**
3.0	0.00	45.94	0.65	2.31	0.03	501.49	58.40	61.49
3.5	0.00	44.15	0.54	2.00	0.01	306.99	46.55	47.00
4.0	0.00	30.58	0.41	1.48	0.00	212.62	34.19	33.95
4.5	0.00	12.52	0.29	0.81	0.01	175.23	25.15	24.69
5.0	0.00	8.33	0.20	0.54	0.00	138.14	18.12	18.34
5.5	0.00	8.81	0.15	0.55	0.01	88.70	12.20	13.01
6.0	0.00	9.58	0.10	0.48	0.00	65.93	6.67	8.42
6.5	0.00	9.73	0.07	0.49	0.00	66.95	3.87	5.39
7.0	0.00	0.00	0.00	0.00	0.00	0.00	0.00	0.00

a) Raman **b)** Orbach **c)** Total

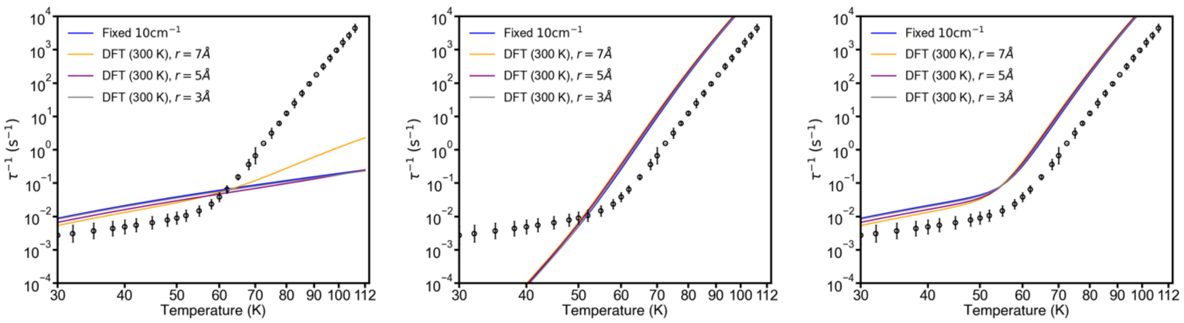

Figure S15. Experimental (black circles) and calculated a) Raman, b) Orbach and c) total magnetic reversal rates for **1** as a function of temperature with a fixed phonon linewidth of $\Gamma = 10\ \mathrm{cm}^{-1}$ (blue) or the calculated mode-dependent linewidths at 300 K obtained with pair-distance cutoffs of $r_{\mathrm{cut}} = 7$ (orange), 5 (purple) and 3 Å (grey). The calculations were performed using the solid-state phonon modes evaluated on a 2×2×2 q-point mesh. Error bars on the experimental rates correspond to one estimated standard deviation.[26,27]

a) Raman **b)** Orbach **c)** Total

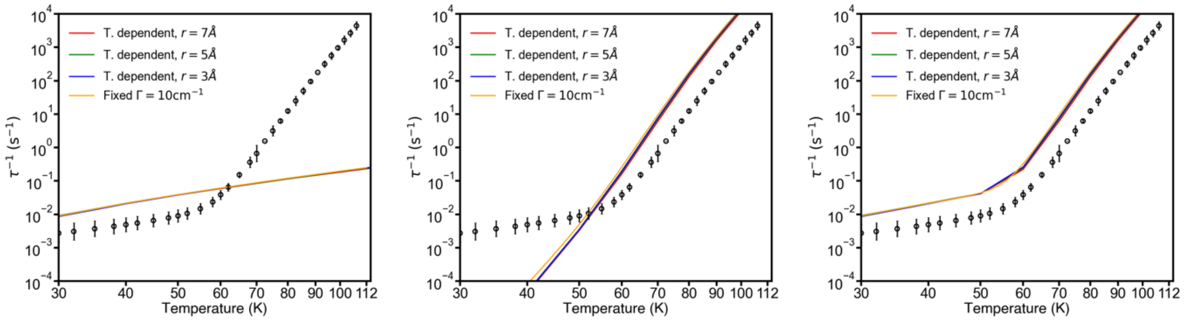

Figure S16. Experimental (black circles) and calculated a) Raman, b) Orbach and c) total magnetic reversal rates for **1** as a function of temperature with mode- and temperature-dependent calculated phonon linewidths obtained with pair-distance cutoffs of $r_{\mathrm{cut}} = 7$ (red), 5 (green) and 3 (blue) Å, or a fixed linewidth of $\Gamma = 10\ \mathrm{cm}^{-1}$ (orange). The calculations were performed using the solid-state phonon modes evaluated on a 2×2×2 q-point mesh. Error bars on the experimental rates correspond to one estimated standard deviation.[26,27]

a) Raman **b)** Orbach **c)** Total

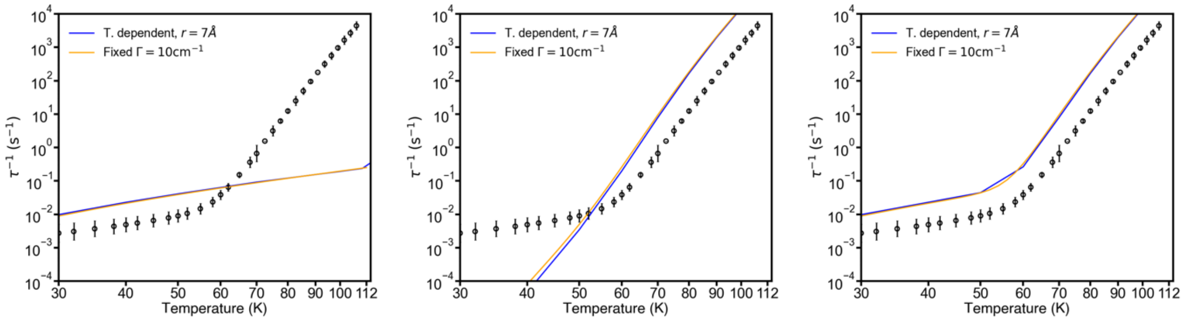

Figure S17. Experimental (black circles) and calculated a) Raman, b) Orbach and c) total magnetic reversal rates for **1** as a function of temperature with mode- and temperature-dependent calculated phonon linewidths obtained with a pair-distance cutoffs of r_{cut} = 7 Å (blue), or a fixed linewidth of Γ = 10 cm^{-1} (orange). The calculations were performed using the solid-state phonon modes evaluated on a 3×3×3 q-point mesh. Error bars on the experimental rates correspond to one estimated standard deviation.[26,27]

a) 13.8 cm^{-1} **b)** 99.1 cm^{-1} **c)** 997.1 cm^{-1}

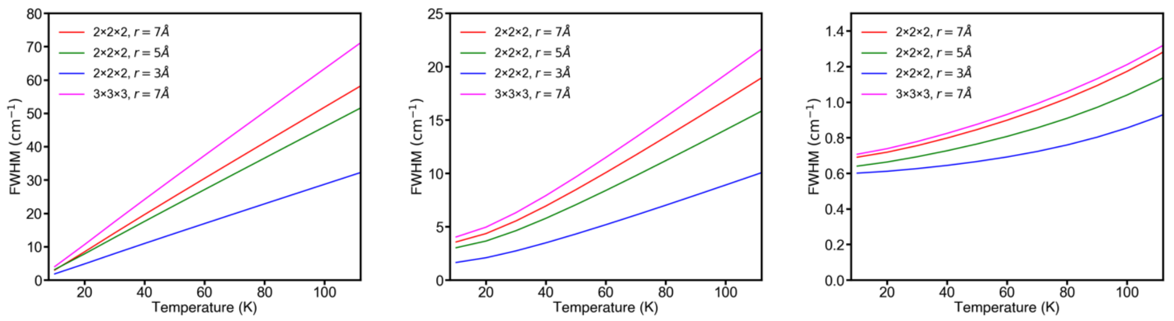

Figure S18. Calculated phonon linewidths as a function for temperature for three different modes (a, b, c), obtained using a range of pair-distance cutoffs and q-point meshes.

References

1 G. Kresse and J. Hafner, *Phys. Rev. B*, 1993, **47**, 558–561.

2 G. Kresse and J. Hafner, *Phys. Rev. B*, 1994, **49**, 14251–14269.

3 G. Kresse and J. Furthmüller, *Comput. Mater. Sci.*, 1996, **6**, 15–50.

4 G. Kresse and J. Furthmüller, *Phys. Rev. B*, 1996, **54**, 11169–11186.

5 J. P. Perdew, K. Burke and M. Ernzerhof, *Phys. Rev. Lett.*, 1996, **77**, 3865–3868.

6 S. Grimme, J. Antony, S. Ehrlich and H. Krieg, *J. Chem. Phys.*, 2010, **132**, 154104.

7 G. Kresse and J. Hafner, *J. Phys. Condens. Matter*, 1994, **6**, 8245–8257.

8 G. Kresse and D. Joubert, *Phys. Rev. B*, 1999, **59**, 1758–1775.

9 V. S. Parmar, A. M. Thiel, R. Nabi, G. K. Gransbury, M. S. Norre, P. Evans, S. C. Corner, J. M. Skelton, N. F. Chilton, D. P. Mills and J. Overgaard, *Chem. Commun.*, 2023, **59**, 2656–2659.

10 A. Togo and I. Tanaka, *Scr. Mater.*, 2015, **108**, 1–5.

11 A. Togo, L. Chaput and I. Tanaka, *Phys. Rev. B*, 2015, **91**, 094306.

12 D. Reta, J. G. C. Kragskow and N. F. Chilton, *J. Am. Chem. Soc.*, 2021, **143**, 5943–5950.

13 J. G. C. Kragskow, A. Mattioni, J. K. Staab, D. Reta, J. M. Skelton and N. F. Chilton, *Chem. Soc. Rev.*, 2023, **52**, 4567–4585.

14 R. Nabi, J. K. Staab, A. Mattioni, J. G. C. Kragskow, D. Reta, J. M. Skelton and N. F. Chilton, *J. Am. Chem. Soc.*, 2023, jacs.3c06015.

15 M. J. Frisch, G. W. Trucks, H. B. Schlegel, G. E. Scuseria, M. A. Robb, J. R. Cheeseman, G. Scalmani, V. Barone, B. Mennucci, G. A. Petersson, H. Nakatsuji, M. Caricato, X. Li, H. P. Hratchian, A. F. Izmaylov, J. Bloino, G. Zheng, J. L. Sonnenberg, M. Hada, M. Ehara, K. Toyota, R. Fukuda, J. Hasegawa, M. Ishida, T. Nakajima, Y. Honda, O. Kitao, H. Nakai, T. Vreven, J. A. Montgomery Jr., J. E. Peralta, F. Ogliaro, M. Bearpark, J. J. Heyd, E. Brothers, K. N. Kudin, V. N. Staroverov, R. Kobayashi, J. Normand, K. Raghavachari, A. Rendell, J. C. Burant, S. S. Iyengar, J. Tomasi, M. Cossi, N. Rega, J. M. Millam, M. Klene, J. E. Knox, J. B. Cross, V. Bakken, C. Adamo, J. Jaramillo, R. Gomperts, R. E. Stratmann, O. Yazyev, A. J. Austin, R. Cammi, C. Pomelli, J. W. Ochterski, R. L. Martin, K. Morokuma, V. G. Zakrzewski,

G. A. Voth, P. Salvador, J. J. Dannenberg, S. Dapprich, A. D. Daniels, Ö. Farkas, J. B. Foresman, J. V. Ortiz, J. Cioslowski and D. J. Fox, .

16 C. M. Breneman and K. B. Wiberg, *J. Comput. Chem.*, 1990, **11**, 361–373.

17 G. Li Manni, I. Fdez. Galván, A. Alavi, F. Aleotti, F. Aquilante, J. Autschbach, D. Avagliano, A. Baiardi, J. J. Bao, S. Battaglia, L. Birnoschi, A. Blanco-González, S. I. Bokarev, R. Broer, R. Cacciari, P. B. Calio, R. K. Carlson, R. Carvalho Couto, L. Cerdán, L. F. Chibotaru, N. F. Chilton, J. R. Church, I. Conti, S. Coriani, J. Cuéllar-Zuquin, R. E. Daoud, N. Dattani, P. Decleva, C. De Graaf, M. G. Delcey, L. De Vico, W. Dobrautz, S. S. Dong, R. Feng, N. Ferré, M. Filatov(Gulak), L. Gagliardi, M. Garavelli, L. González, Y. Guan, M. Guo, M. R. Hennefarth, M. R. Hermes, C. E. Hoyer, M. Huix-Rotllant, V. K. Jaiswal, A. Kaiser, D. S. Kaliakin, M. Khamesian, D. S. King, V. Kochetov, M. Krośnicki, A. A. Kumaar, E. D. Larsson, S. Lehtola, M.-B. Lepetit, H. Lischka, P. López Ríos, M. Lundberg, D. Ma, S. Mai, P. Marquetand, I. C. D. Merritt, F. Montorsi, M. Mörchen, A. Nenov, V. H. A. Nguyen, Y. Nishimoto, M. S. Oakley, M. Olivucci, M. Oppel, D. Padula, R. Pandharkar, Q. M. Phung, F. Plasser, G. Raggi, E. Rebolini, M. Reiher, I. Rivalta, D. Roca-Sanjuán, T. Romig, A. A. Safari, A. Sánchez-Mansilla, A. M. Sand, I. Schapiro, T. R. Scott, J. Segarra-Martí, F. Segatta, D.-C. Sergentu, P. Sharma, R. Shepard, Y. Shu, J. K. Staab, T. P. Straatsma, L. K. Sørensen, B. N. C. Tenorio, D. G. Truhlar, L. Ungur, M. Vacher, V. Veryazov, T. A. Voß, O. Weser, D. Wu, X. Yang, D. Yarkony, C. Zhou, J. P. Zobel and R. Lindh, *J. Chem. Theory Comput.*, 2023, **19**, 6933–6991.

18 M. Reiher, *Theor. Chem. Acc.*, 2006, **116**, 241–252.

19 F. Aquilante, L. Gagliardi, T. B. Pedersen and R. Lindh, *J. Chem. Phys.*, 2009, **130**, 154107.

20 B. O. Roos, R. Lindh, P.-Å. Malmqvist, V. Veryazov and P.-O. Widmark, *J. Phys. Chem. A*, 2005, **109**, 6575–6579.

21 B. O. Roos, R. Lindh, P.-Å. Malmqvist, V. Veryazov and P.-O. Widmark, *J. Phys. Chem. A*, 2004, **108**, 2851–2858.

22 J. K. Staab and N. F. Chilton, *J. Chem. Theory Comput.*, 2022, **18**, 6588–6599.

23 J. Staab and N. Chilton, *J. Chem. Theory Comput.*, 2024, **20**, 2969–2970.

24 B. A. Heß, C. M. Marian, U. Wahlgren and O. Gropen, *Chem. Phys. Lett.*, 1996, **251**, 365–371.

25 L. A. Mariano, S. Mondal and A. Lunghi, *J. Chem. Theory Comput.*, 2024, **20**, 323–332.

26 D. Reta and N. F. Chilton, *Phys. Chem. Chem. Phys.*, 2019, **21**, 23567–23575.

27 W. J. A. Blackmore, G. K. Gransbury, P. Evans, J. G. C. Kragskow, D. P. Mills and N. F. Chilton, *Phys. Chem. Chem. Phys.*, , DOI:10.1039/D3CP01278F.

28 A. Lunghi, F. Totti, R. Sessoli and S. Sanvito, *Nat. Commun.*, 2017, **8**, 14620.
